# Important to Medical Students

**Published:** 1891-09

**Authors:** 


### IMPORTANT TO MEDICAL STUDENTS.

THE Cleveland *Medical Gazette* for August, 1891, publishes some good advice under the above title, which we cordially endorse and cheerfully reproduce :

"Several medical colleges are advertising to graduate students at the end of the coming Winter term upon the attendance of *two courses of lectures.* Medical students should be informed that graduates of such schools will not be entitled to practice medicine in more than half the States of the Union, representing over forty millions inhabitants.

"The State Examining Boards throughout the country have given sufficient notice of what would be required of medical colleges, and all reputable schools have adopted the three-year graded course of study; but several so-called medical colleges throughout the country thinking to profit by the increased time of study required by most medical schools, are soliciting patronage by offering to graduate *one more* class at the end of the second course of lectures. We believe it to be the duty of the medical journals to warn students against all such institutions and decline to insert advertisements of these schools. This will be our policy. We had hoped to publish a list of all schools not coming up to the requirements of the various State Boards of Health, but have failed to secure the complete list for this number of the *Gazette.*"

